# Overview on WHO-HAEM5 and the diagnostic relevance of genetic alterations for the classification

**DOI:** 10.1515/medgen-2024-2008

**Published:** 2024-03-06

**Authors:** Claudia Haferlach, German Ott, Katharina Hörst, Constanze Kühn, Torsten Haferlach, Reiner Siebert

**Affiliations:** MLL – Munich Leukemia Laboratory Munich Germany; MLL – Munich Leukemia Laboratory Munich Germany; MLL – Munich Leukemia Laboratory Munich Germany; MLL – Munich Leukemia Laboratory Munich Germany; Robert-Bosch-Hospital Department for Clinical Pathology Stuttgart Germany; Ulm University Ulm University Medical Center Ulm Germany

## Abstract

The landscape of haematological malignancies is constantly evolving, driven by advances in our understanding of their genetic basis. This has cumulated within the 5^th^ Edition of the World Health Organization (WHO) Classification of Haematolymphoid Tumours published in short form in 2022 [1, 2] and being available in full length both as “Blue Book” (in print expected early 2024) as well as web-based classification (see: https://tumourclassification.iarc.who.int/welcome/). Similarly, the importance of genetic alterations for the classification is highlighted in other classification systems related to haematologic neoplasms [3–5]. In this special issue of the Medizinische Genetik, we present a comprehensive overview of the genetic alterations contributing to the classification of haematolymphoid neoplasms in the 5^th^ Edition of the WHO classification (WHO-HAEM5) and its diagnostic relevance in the context of various haematological malignancies.

Keywords: WHO, classification, overview, genetics, haematolymphoid tumours

## The WHO-HAEM5 classification system: a genetic paradigm shift

For myeloid neoplasms in particular, the introduction of the WHO-HAEM5 classification system in 2022 represents a turning point in the field of hematologic oncology. In contrast to its predecessors, this system takes a genetic-centric approach that recognizes the paramount importance of genetic alterations in the classification of diseases. By incorporating genetic markers as integral diagnostic criteria, WHO-HAEM5 provides a refined framework for characterizing haematological malignancies. It not only enhances diagnostic precision but also offers valuable insights into disease prognosis and guides therapeutic decisions.

**Figure 1:**
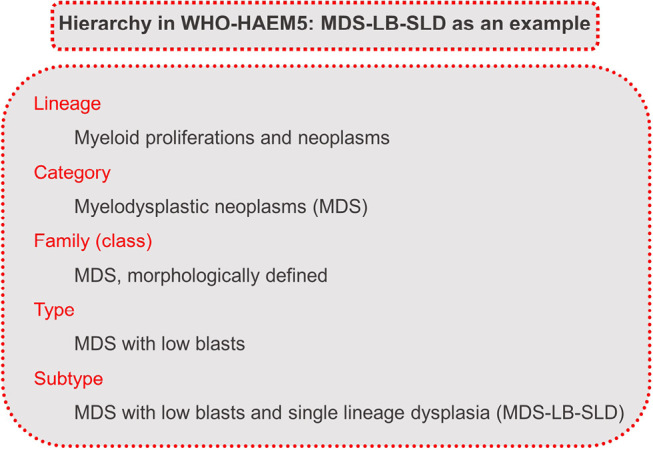
Classification of myelodysplastic neoplasm with low blasts and single lineage dysplasia (MDS-LB-SLD) in WHO-HAEM5 as an example to illustrate the hierarchy of the classification [6].

The framework of WHO-HAEM5 includes a hierarchy from benign to malignant, with categories such as lineage, family, type, and subtype as depicted in an example (figure 1). The classification utilizes a triad of attributes: lineage, dominant clinical attribute, and dominant biologic attribute. The first part of WHO-HAEM5 focuses on myeloid neoplasms, emphasizing advancements in understanding their biology. Histiocytic/dendritic neoplasms are grouped based on their unique lineage. WHO-HAEM5 also addresses preneoplastic diseases and tumour-like lesions resembling B-cell and T-cell neoplasms. Lymphoblastic leukaemias/lymphomas are categorized by lineage, covering a broad range of diseases, and attempts to separate T-cell and NK-cell neoplasms were considered but deemed challenging in routine practice. In accordance with to WHO-HAEM5, a separate Blue Book on Genetic Tumour Syndromes will be published.

## Appraisal of current technologies for genetic exploration

Central to the implementation of the WHO-HAEM5 is our ability to harness cutting-edge genetic technologies. These technologies empower us to comprehensively profile the genetic landscape of haematological malignancies, uncover small nucleotide variants, copy number alterations, and structural variants. These techniques have become indispensable tools to identify the genetic alterations crucial for the WHO-HAEM5 classification.

### Practical guides to genetic studies / new and modified categories in WHO-HAEM5

With the updated version, WHO-HAEM5 has introduced various changes to the classification and also added new entities. From risk stratification to treatment decisions, genetics is at the heart of the benefits of WHO-HAEM5.

**Figure 2:**
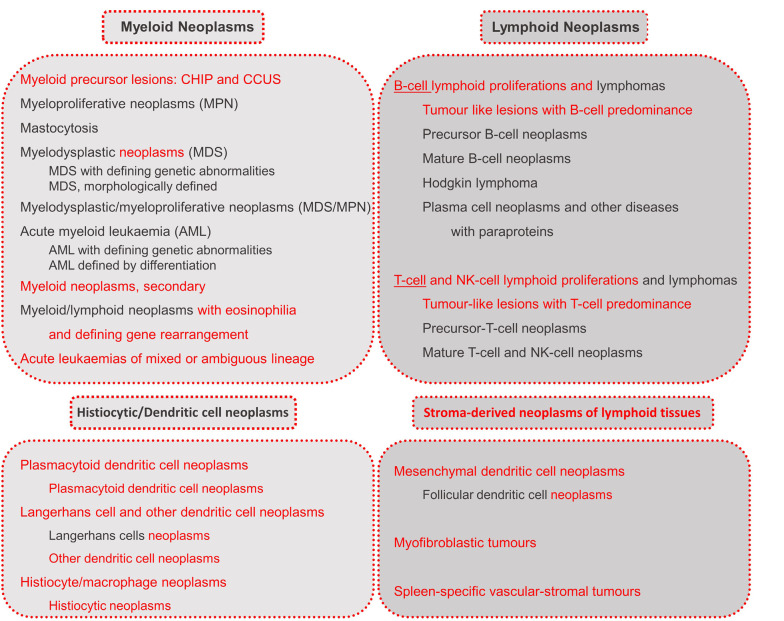
Categories of haematolymphoid neoplasms according to WHO-HAEM5 [6]. New features in WHO-HAEM5 are marked in red.

For a first overview, the different categories of myeloid and lymphoid neoplasms are listed in figure 2.

In the WHO-HAEM5, where possible, a triad of attributes was systematically applied and included: lineage + dominant clinical attribute + dominant biologic attribute. The latter encompasses gene fusions, rearrangements, and mutations. Gene fusions are part of the nomenclature of types/subtypes when the identities of both implicated genes are required or represent desirable criteria for diagnosis (e. g., *PML*::*RARA*). According to the latest notation for gene fusions, the involved genes are separated by a double colon [6, 7].

The first part of the WHO-HAEM5 is dedicated to myeloid neoplasms, the biology of which has been better elucidated, while their treatment remains as a whole an unmet need despite advances [1].

### The disease continuum of clonal haematopoiesis, myelodysplastic neoplasms (MDS), and acute myeloid leukaemia

**Figure 3:**
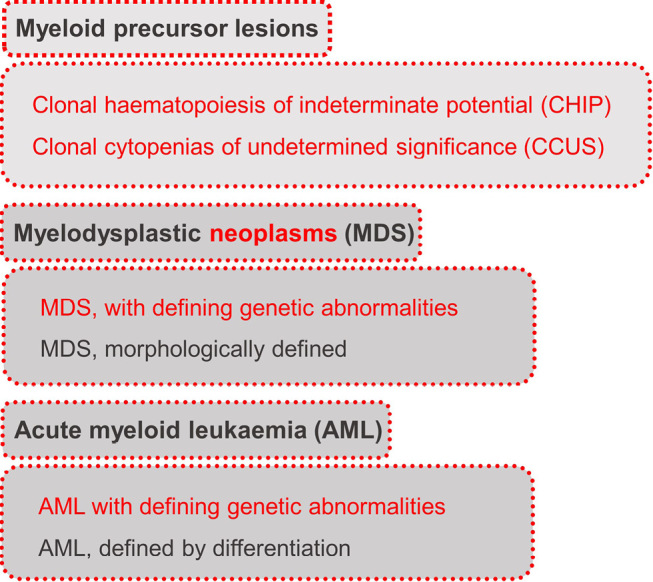
Disease continuum of Clonal Haematopoiesis, Myelodysplastic Neoplasms (MDS), and Acute Myeloid Leukaemia according to WHO-HAEM5 [6]. New features in WHO-HAEM5 are marked in red.

The understanding of clonal haematopoiesis (CH), myelodysplastic neoplasms (MDS) and acute myeloid leukaemia (AML) has witnessed a paradigm shift in recent years and this group is now seen as a disease continuum rather than completely separate entities (figure 3). CH is for the first time classified as a myeloid precursor lesion in WHO-HAEM5. Its hallmark, the clonal expansion of a single hematopoietic cell, is the basis for both clonal haematopoiesis of indeterminate potential (CHIP) and clonal cytopenia of undetermined significance (CCUS). The transition to MDS occurs with increasing genetic complexity, but also with morphological changes such as dysplasia [6]. The increasing influence of genetic parameters in diagnostics can be seen in the classification of MDS in WHO-HAEM5: MDS is grouped into three subtypes with defining genetic abnormalities (MDS-5q, MDS-*SF3B1* and MDS-bi*TP53*) next to other, morphologically defined subtypes [6]. MDS is associated with an increased risk of transformation to AML. Similar to MDS, genetic subtypes also play a pivotal role in the classification of AML. In WHO-HAEM5, AML is divided into AML, defined by differentiation and AML with defining genetic abnormalities. The latter encompasses AML subtypes with various underlying gene fusions, rearrangements or mutations [6]. In AML diagnostics, a blast count of at least 20 % was for many years a decisive criterion. Already in the previous WHO version published in 2017 (WHO-HAEM4R), a subset of myeloid neoplasms harbouring a distinct genetic abnormality were classified as AML irrespective of blast count. The number of genetic abnormalities defining AML irrespective of blast count was increased in WHO-HAEM5, underlining that genetic aberrations are more relevant for classification than the blast count. This is also evident in various entity names that are based on genetic aberrations [6, 8]. The elimination of the blast cut-off in the presence of clearly defined genetic alterations underlines the importance of distinct genetic changes.

### Genetic alterations in myeloproliferative neoplasms and MDS/MPN

**Figure 4:**
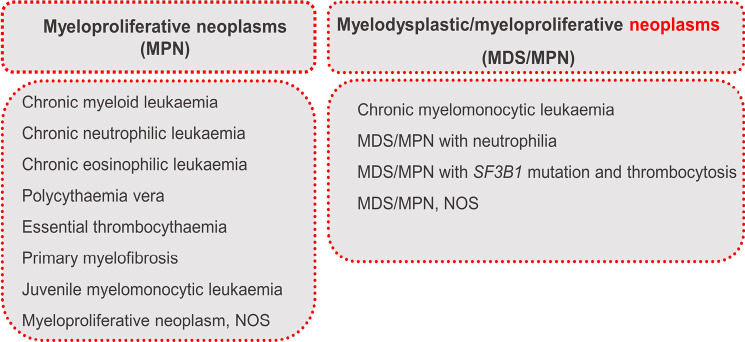
Myeloproliferative Neoplasms and MDS/MPN according to WHO-HAEM5 [6]. New features in WHO-HAEM5 are marked in red.

Myeloproliferative neoplasms (MPN) and myelodysplastic neoplasms/myeloproliferative neoplasm (MDS/MPN) entities represent a heterogeneous group of haematological disorders characterized by clonal expansion and dysregulation of myeloid cells. Within the myeloid neoplasms, the distinction between MDS with its characteristic cytopenia on the one hand and MPN with cytosis on the other hand is of crucial importance in diagnostics. What makes the diagnosis more difficult are MDS/MPN entities, as these have characteristics of both MDS and MPN. Genetically, MPNs are distinguished by the presence or absence of a *BCR*::*ABL1*-rearrangement [6]. Chronic myeloid leukaemia (CML) as the only *BCR*::*ABL1*-positive MPN may harbor, besides its characteristic rearrangement, additional chromosomal aberrations having prognostic impact [9]. Of note, CML is now always *BCR*::*ABL1* positive – the term MDS/MPN with neutrophilia replaces the old designation atypical CML. The classic *BCR*::*ABL1*-negative MPNs, polycythemia vera (PV), essential thrombocythemia (ET), and primary myelofibrosis (PMF), share an overactive *JAK2*-signaling as a common characteristic with the key driver genes *JAK2*, *CALR*, and *MPL*. Apart from these classic *BCR*::*ABL1*-negative MPNs, there are various other rare MPNs with unique genetic features. Between MDS and MPNs, MDS/MPN find their place with overlapping features of both entities. These include MDS/MPN-*SF3B1*-T, which impressively demonstrates the intermediate position between MDS and MPN: MDS/MPN-*SF3B1*-T has a characteristic *SF3B1* mutation similar to the MDS subtype MDS-*SF3B1* and concurrently harbours *JAK2* mutations as found in MPN [6]. MPN and MDS/MPN are displayed in figure 4.

### Myeloid/lymphoid neoplasms with eosinophilia and defining gene rearrangement and acute leukaemias of mixed or ambiguous lineage

**Figure 5:**
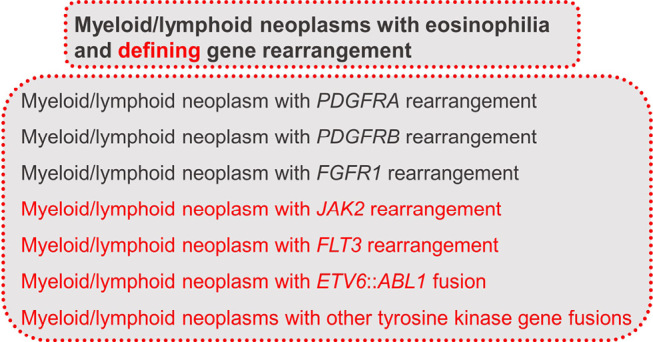
Overview of myeloid/lymphoid neoplasms with eosinophilia and defining gene rearrangements according to WHO-HAEM5 [6]. New features in WHO-HAEM5 are marked in red.

In WHO-HAEM5, the group of “Myeloid/lymphoid neoplasms with eosinophilia and defining gene rearrangements” comprises diseases caused by dysregulated fusion tyrosine kinase (TK) genes. Within this group, patients can present with MDS/MPN as well as de novo or secondary mixed-phenotype leukaemias or lymphomas in addition to MPN. Eosinophilia is present often. The emerging importance of genetics is recognizable in the title of the group, where “defining” has been inserted in WHO-HAEM5. The entities are listed in figure 5 [6].

Genetics also plays an extremely important role in therapy. The response to tyrosine kinase inhibitors is dependent on the defining genetic aberration [6].

**Figure 6:**
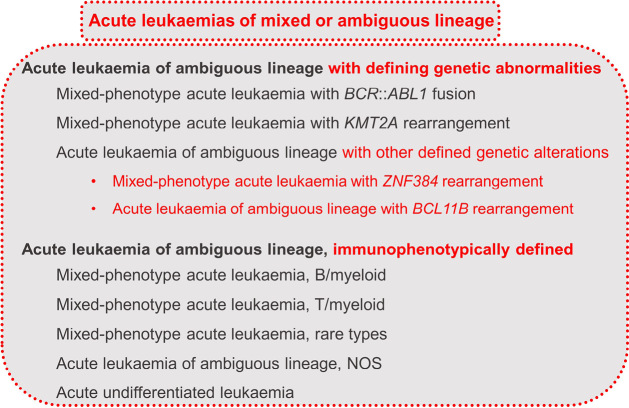
Overview of acute leukaemias of mixed or ambiguous lineage according to WHO-HAEM5 [6]. New features in WHO-HAEM5 are marked in red.

Acute leukaemias of mixed or ambiguous lineage (ALAL) show ≥20 % abnormal progenitor cells that do not differentiate along one lineage. An increasing variety of molecular and genetic abnormalities have been described. ALALs include the entities listed in figure 6 [6].

### Histiocytic/Dendritic cell neoplasms and stroma-derived neoplasms of lymphoid tissues

Histiocytic and dendritic cell neoplasms are tumours originating from cells of the mononuclear phagocyte system, which includes monocytes, macrophages, and dendritic cells. The neoplastic counterparts are various, including histiocytic sarcoma, Rosai-Dorfman disease, and more. These neoplasms often exhibit immunophenotypes corresponding to their normal counterparts. Some of these neoplasms arise de novo, while others are associated with or follow a preceding lymphoma or leukaemia. These secondary neoplasms are classified similarly to their de novo counterparts and may share clonal markers with the associated or preceding lymphoma [6].

**Figure 7:**
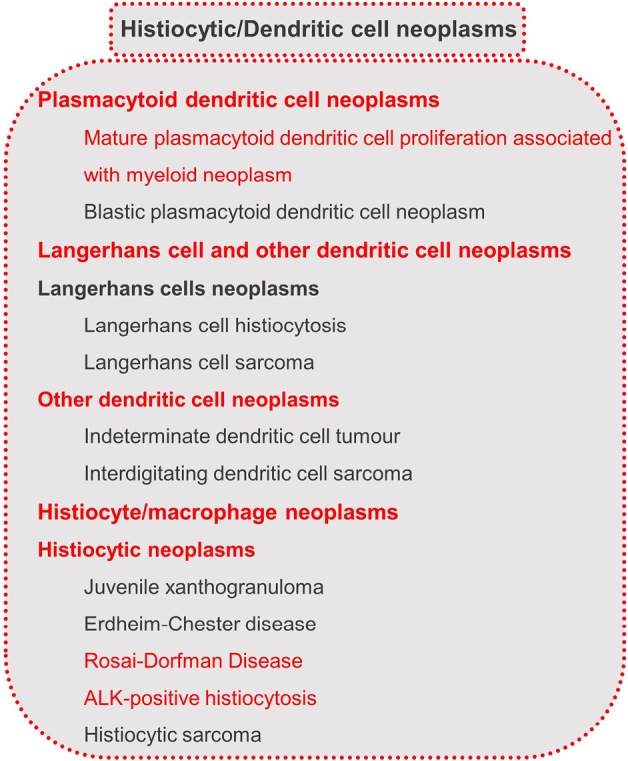
Histiocytic/Dendritic cell neoplasms according to WHO-HAEM5 [6]. New features in WHO-HAEM5 are marked in red.

Within the histiocytic/dendritic cell neoplasms, “blastic plasmacytoid dendritic cell neoplasm (BPDCN)” has been added to the group of “plasmacytoid dendritic cell neoplasms”, and the entities “mature plasmacytoid dendritic cell proliferation associated with myeloid neoplasm (MPDCP)”, “Rosai-Dorfman disease”, and “ALK-positive histiocytosis” were newly included. MPDCP may encompass clonal processes, and Rosai-Dorfman disease often includes gain-of-function mutations in the MAPK pathway. The neoplastic nature of these entities provides a rationale for their inclusion in WHO-HAEM5 [6]. Overall, the entities belonging to the section of “Histiocytic/Dendritic cell neoplasms” are listed in figure 7 [6].

**Figure 8:**
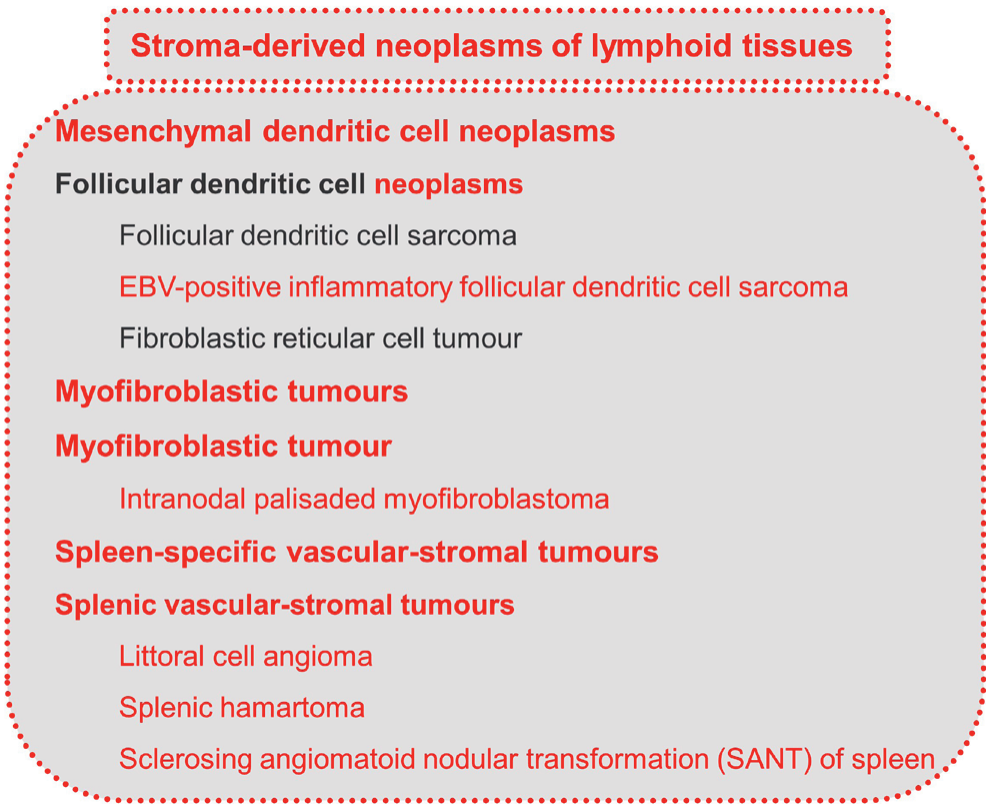
Stroma-derived neoplasms of lymphoid tissues according to WHO-HAEM5 [6]. New features in WHO-HAEM5 are marked in red.

The latest classification changes in WHO-HAEM5 include moving “follicular dendritic cell sarcoma” and “fibroblastic reticular cell tumour” to a separate category of “stroma-derived neoplasms of lymphoid tissues”. This category is a new addition in WHO-HAEM5 and includes mesenchymal tumours specific to lymph node and spleen. Since follicular dendritic cells and fibroblastic reticular cells are of mesenchymal origin and are not derived from haematopoietic stem cells, the entities were moved from the category of “Histiocytic/Dendritic cell neoplasms” to the new category. The section of “Stroma-derived neoplasms of lymphoid tissues” includes the entities listed in figure 8 [6]:

### Genetic alterations in acute lymphoblastic leukaemias/lymphomas

The various and genetically diverse myeloid neoplasms are distinguished from lymphoid neoplasms, which also exhibit a variety of genetic peculiarities. According to WHO-HAEM5, lymphoblastic leukaemia (ALL)/lymphoblastic lymphoma (LBL), is assigned to the lymphoid precursor neoplasms of the B- or T-cell type. By convention, the term leukaemia (ALL) is used when peripheral blood and bone marrow are the primary site of involvement, and the term lymphoma (LBL) when the primary involvement is at lymph nodes or extra-nodal sites [6]. For B-ALL/LBL diagnosis, a combination of cytomorphology and immunophenotyping is required. However, for classification of B-ALL/LBL in WHO-HAEM5, genetic abnormalities form the fundamental basis leading to various genetic subtypes. Some of the entities can be defined by traditional karyotype or FISH analysis, but many of the newer entities require NGS or transcriptomics to identify defining, often cytogenetically cryptic abnormalities. Therefore, a combination of genetic techniques is needed for comprehensive classification and risk stratification of B-ALL/LBL. In T-ALL/LBL, various chromosomal aberrations may occur, the most common alterations leading to aberrant expression of oncogenic transcription factors. Based on the oncogene involved, T-ALL/LBLs are classified into four distinct molecular groups (*TAL* or *LMO*, *TLX1*, *TLX3*, *HOXA*). In addition, mutations in several genes are recognized including activating mutations in the *NOTCH1* gene in over 50 % of T-ALL cases [6].

### Genetic alterations in chronic lymphocytic leukaemia and plasma cell neoplasms

Within the mature B-cell neoplasms, monoclonal B-cell lymphocytosis (MBL) and chronic lymphocytic leukaemia/small lymphocytic lymphoma (CLL/SLL) are now assigned to the family/class of “pre-neoplastic and neoplastic small lymphocytic proliferations” in WHO-HAEM5. In the 2022 classification, the subgroups for MBL were adjusted into low-count MBL or clonal B-cell expansion, CLL/SLL-type MBL and non-CLL/SLL-type MBL instead of the categories CLL-type, atypical CLL-type and non-CLL-type. For CLL, *TP53* mutational analysis, *IGHV* region somatic hypermutation analysis and B-cell receptor stereotype subset analysis are now considered essential for full prognostic evaluation [1, 6].

The section of plasma cell neoplasms and other diseases with paraproteins was reorganized in WHO-HAEM5 based on types of paraproteins and disease burden. Therefore, IgM and non-IgM monoclonal gammopathy of undetermined/renal significance are grouped as monoclonal gammopathies and the diseases with abnormal monoclonal immunoglobulin deposits are grouped together [6]. Plasma cell neoplasms in WHO-HAEM5 include plasmacytoma, plasma cell myeloma (PCM)/multiple myeloma (MM) and plasma cell neoplasms with associated paraneoplastic syndrome. Although the recurrent immunoglobulin translocations in PCM/MM are mutual exclusive and some of them are associated with a more favourable or unfavourable outcome, current knowledge on the underlying biology is limited. Therefore, there is no molecular subclassification in PCM/MM [6].

Within the entity of plasma cell neoplasms with associated paraneoplastic syndrome, TEMPI syndrome is now confirmed as subtype of plasma cell neoplasms, after being a provisional entity in WHO-HAEM4. Furthermore, AESOP syndrome (adenopathy and extensive skin patch overlying a plasmacytoma) was included in the classification as a subtype. Non-IgM monoclonal gammopathy of undetermined significance is grouped with other monoclonal gammopathies in WHO-HAEM5 and diseases characterised by monoclonal immunoglobulin deposition such as immunoglobulin-related (AL) amyloidosis, and monoclonal immunoglobulin deposition disease, are grouped separately in WHO-HAEM5. Staging of multiple myeloma by the R-ISS (revised international staging system for multiple myeloma) is recommended by WHO-HAEM5 as strong predictor of survival. In addition, minimal/measurable residual disease (MRD) assessment has gained significant importance for multiple myeloma [6].

### Genetic alterations in mature B- and T-cell lymphomas

In WHO-HAEM5, the category of mature B-cell neoplasms comprises 12 families/classes. One important change within those categories affect splenic B-cell lymphomas and leukaemias, within which the term “splenic B-cell lymphoma/leukaemia with prominent nucleoli” replaces the provisional category of “hairy cell leukaemia (HCL) variant (v)” of WHO-HAEM4R and also absorbs cases of “CD5-negative B-cell prolymphocytic leukaemia”, among others. This adaption has been done based on the now recognized biological differences between HCLv and HCL, although the morphology might be similar. The differences include genetic factors like the absence vs presence of the *BRAF* p.V600E mutation [6].

Another major change in WHO-HAEM5 concerns follicular lymphoma (FL). Lymphomas with a follicular growth pattern, carrying a translocation involving the *BCL2* gene, and harbouring centrocytes and centrobasts are now termed classic FL (cFL), thereby set apart from less frequent subtypes. Of importance, grading of FL is no longer considered mandatory in WHO-HAEM5 due to the poor reproducibility and the absence of significant differences in clinical outcome. On the basis of rearrangements, FL can be divided into the groups of “FL with *BCL2* rearrangement (*BCL2*-R FL)”, “FL with *BCL6* rearrangement (*BCL6*-R FL)” and “FL lacking *BCL2* and *BCL6* rearrangements” [6].

In addition, important adaptations have also been made in the previous category of high-grade B-cell lymphomas with dual rearrangements of *MYC* and *BCL2* and/or *BCL6* of the WHO-HAEM4R. Taking into account their variable morphology but homogeneous biological features and gene expression patterns, “double hit” cases with dual *MYC* and *BCL2* rearrangements are designated diffuse large B-cell lymphoma/high-grade B-cell lymphoma with *MYC* and *BCL2* rearrangements (DLBCL/HGBL-*MYC*/*BCL2*). Aggressive lymphoid neoplasms with dual *MYC* and *BCL6* rearrangements are now classified as genetic subtypes of either DLBCL, NOS or HGBL, NOS according to their cytomorphological features. The former Burkitt-like lymphoma with 11q aberration is renamed high-grade B-cell lymphoma with 11q aberrations (HGBL-11q) in WHO-HAEM5. This aggressive lymphoma shows a mutational spectrum that is different from that of Burkitt lymphoma and resembles more the spectrum of DLBCL [1, 6].

In peripheral T-cell lymphoma, not otherwise specified, (PTCL, NOS) two molecular subtypes have been identified with the help of gene expression profiling studies: PTCL-GATA3 and PTCL-TBX21, characterized by overexpression of the transcription factors *TBX21* or *GATA3* [6].

## Unraveling germline predispositions

Genetic alterations beyond somatic mutations and chromosomal aberrations are relevant in haematology, thus WHO-HAEM5 encompasses also germline mutations predisposing to haematological neoplasms. Germline predisposition to hematologic malignancies is an evolving field that has gained significant attention in recent years. Understanding these germline predispositions is not only essential for early detection but also has profound implications for genetic counselling and family-based screening. WHO-HAEM5 incorporates key genetic highlights and changes that are crucial for geneticists, as they help to elucidate the genetic landscape underlying these conditions, and proposes features which should indicate germline genetic work-up:

Diagnosis of an entity (or features) of haematolymphoid neoplasms typically associated with germline predisposition according to WHO-HAEM5;
Known germline-predisposition for cancer in a family;
Fulfilment of diagnostic criteria for any established cancer predisposition syndrome, like Li Fraumeni Syndrome, hereditary breast-ovarian cancer, familial colorectal cancer, etc.;
Occurrence of a haematolymphoid malignancy in a patient with syndromic features (e. g. dysmorphisms, organ malformations, developmental disorder);
Diagnosis of a haematolymphoid neoplasm in two first-or second-degree relatives in a family;
Diagnosis of two independent haematolymphoid neoplasms in the same person or a (non-therapy induced) haematolymphoid neoplasms in a person with multiple or early-onset solid tumours;
Increased and unexplained treatment toxicity in a patient with haematolymphoid neoplasms;
Very early-onset of a haematolymphoid neoplasm as compared to the general epidemiology of the disease;
Detection of a pathogenic or likely pathogenic variant in the somatic work-up of a neoplasm, if the variant affects a gene associated to germline cancer predisposition and sequencing findings are compatible if a germline origin.

**Figure 9:**
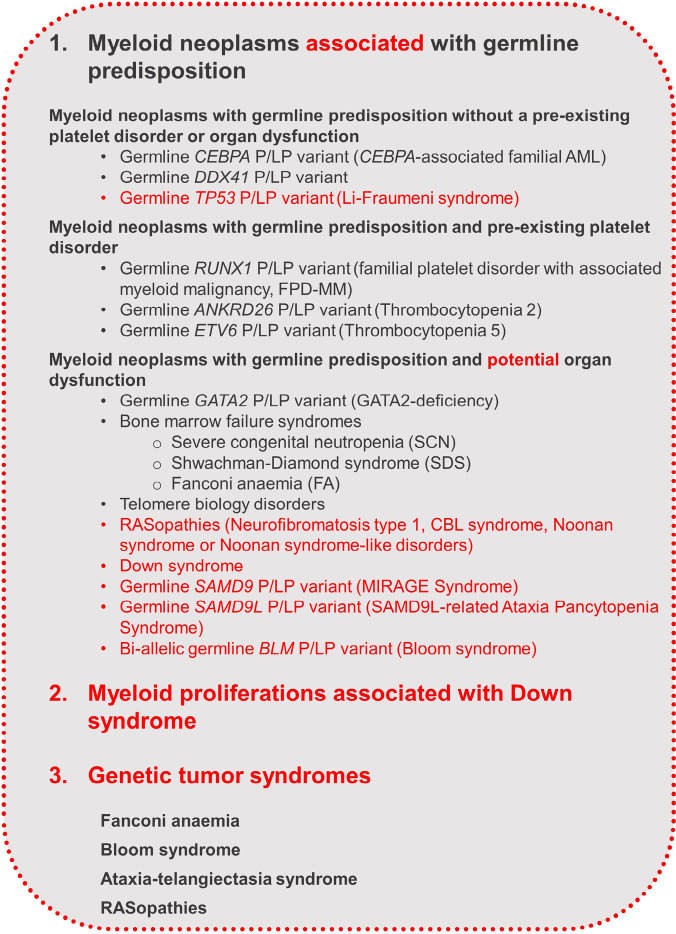
Overview of the germline conditions classified in WHO HAEM5 [6]. New features in WHO-HAEM5 are marked in red.

In WHO-HAEM5, one section is dedicated to myeloid neoplasms associated with germline predispositions which is subdivided into 3 sections: 1. Myeloid neoplasms with germline predisposition without a pre-existing platelet disorder or organ dysfunction (germline mutations in *CEBPA*, *DDX41*, *TP53*), 2. Myeloid neoplasms with germline predisposition and pre-existing platelet disorder (germline mutations in *RUNX1*, *ANKRD26*, *ETV6*), and 3. Myeloid neoplasms with germline predisposition and potential organ dysfunction (e. g.: bone marrow failure syndromes, RASopathies, Fanconi Anaemia). Special attention is given to myeloid proliferations in individuals with Down syndrome. The classification is scalable to include future germline predispositions [6]. Figure 9 provides an overview of the germline conditions classified separately in WHO-HAEM5.

Lymphoid neoplasms associated with clinical syndromes are identified separately, including ataxia telangiectasia (AT) and Nijmegen breakage syndrome (NBS) with specific germline mutations in *ATM* and *NBN*, respectively. Detection of such syndromes is clinically significant, both for treatment planning and for monitoring and counselling affected individuals and their families. Leukaemias and lymphomas are referred to as “AT-related” or “NBS-related” accordingly [1]. Moreover, the classification of immune deficiency and dysfunction associated lymphomas has been vastly restructured, among others to be more closely adherent to the classification of inborn errors of immunity (IEI) caused by damaging germline variants in single genes as proposed by the International Union of Immunological Societies Expert Committee.

Germline tests for monogenic predisposition should also be considered for familial lymphatic entities such as B-ALL/LBL. These may be associated with mutations in *PAX5*, *ETV6* and *TP53* and probable multigenic risk variants (e. g. in *GATA3*, *ARID5B*, *IKZF1*, *CEBPE*, *CDNK2A* or *CDNK2B*) [6].

For the first time, a WHO classification of Genetic Tumour Syndromes will be part of the 5^th^ Edition of the WHO classifications (see: https://tumourclassification.iarc.who.int/welcome/#). This will introduce an organization of the taxonomy of genetic tumour syndromes according to the major mechanism affected and molecular pathways involved, rather than according organ specificity.

## Conclusion: the rising star in haematological malignancies: genetics!

In summary, the WHO-HAEM5 classification system holds a transformative influence in haematological oncology. By placing genetics at its core, WHO-HAEM5 heralds a new era of precision medicine, where diagnosis and treatment are tailored to the unique genetic profiles of each patient.

## References

[1] Alaggio R (2022). The 5th edition of the World Health Organization Classification of Haematolymphoid Tumours: Lymphoid Neoplasms. Leukemia. 36(7).

[2] Khoury J D (2022). The 5th edition of the World Health Organization Classification of Haematolymphoid Tumours: Myeloid and Histiocytic/Dendritic Neoplasms. Leukemia. 36(7).

[3] Arber D A (2022). International Consensus Classification of Myeloid Neoplasms and Acute Leukemias: integrating morphologic, clinical, and genomic data. Blood. 140(11).

[4] Döhner H (2022). Diagnosis and management of AML in adults: 2022 recommendations from an international expert panel on behalf of the ELN. Blood. 140(12).

[5] Campo E (2022). The International Consensus Classification of Mature Lymphoid Neoplasms: a report from the Clinical Advisory Committee. Blood. 140(11).

[6] WHO Classification of Tumours Editorial Board. Haematolymphoid tumours. WHO classification of tumours series 2022; 5th ed.; vol. 11.

[7] Bruford E A (2021). HUGO Gene Nomenclature Committee (HGNC) recommendations for the designation of gene fusions. Leukemia. 35(11).

[8] Swerdlow S H (2017). WHO Classification of Tumours of Haematopoietic and Lymphoid Tissues. WHO Classification of Tumours of Haematopoietic and Lymphoid Tissues.

[9] Hochhaus A (2020). European LeukemiaNet 2020 recommendations for treating chronic myeloid leukemia. Leukemia. 34(4).

